# Dynamics of Visual Perceptual Decision-Making in Freely Behaving Mice

**DOI:** 10.1523/ENEURO.0161-21.2022

**Published:** 2022-03-15

**Authors:** Wen-Kai You (游文愷), Shreesh P. Mysore

**Affiliations:** 1 Department of Psychological and Brain Sciences, Johns Hopkins University, Baltimore, MD 21205; 2 The Solomon H. Snyder Department of Neuroscience, Johns Hopkins University, Baltimore, MD 21205

**Keywords:** decision-making, dynamics, freely-behaving, mouse, perceptual, visual

## Abstract

The temporal dynamics of perceptual decisions offer a key window into the cognitive processes contributing to decision-making. Investigating perceptual dynamics in a genetically tractable animal model can facilitate the subsequent unpacking of the underlying neural mechanisms. Here, we investigated the time course as well as fundamental psychophysical constants governing visual perceptual decision-making in freely behaving mice. We did so by analyzing response accuracy against reaction time (RT), i.e., conditional accuracy, in a series of two-alternative forced choice (2-AFC) orientation discrimination tasks in which we varied target size, luminance, duration, and presence of a foil. Our results quantified two distinct stages in the time course of mouse visual decision-making: a “sensory encoding” stage in which conditional accuracy exhibits a classic trade-off with response speed, and a subsequent “short-term memory (STM)-dependent” stage in which conditional accuracy exhibits a classic asymptotic decay following stimulus offset. We estimated the duration of visual sensory encoding as 200–320 ms across tasks, the lower bound of the duration of STM as ∼1700 ms, and the briefest duration of visual stimulus input that is informative as ≤50 ms. Separately, by varying stimulus onset delay, we demonstrated that the conditional accuracy function (CAF) and RT distribution can be independently modulated, and found that the duration for which mice naturally withhold from responding is a quantitative metric of impulsivity. Taken together, our results establish a quantitative foundation for investigating the neural circuit bases of visual decision dynamics in mice.

## Significance Statement

This study presents a quantitative breakdown of the time course of visual decision-making in mice during naturalistic behavior. It demonstrates parallel stages in mouse visual perceptual decision dynamics to those in humans, estimates their durations, and shows that mice are able to discriminate well under challenging visual conditions, with stimuli that are brief, low luminance, and small. These results set the stage for investigating the neural bases of visual perceptual decision dynamics and their dysfunction in mice.

## Introduction

Exploring the temporal dynamics of perceptual decisions from onset of the sensory input through the initiation of behavioral responses affords a key window into the underlying cognitive processes (Uchida et al., 2006; Stanford et al., 2010; Siegel et al., 2011). Investigations of such dynamics in humans (Steinemann et al., 2018; Wilming et al., 2020) and other species (Yang et al., 2008; Zariwala et al., 2013; Thura and Cisek, 2014) have revealed distinct stages in perceptual processing, their timing, and their interactions (Wickelgren, 1977; McElree and Dosher, 1989; Heitz, 2014). Performing such investigations in a genetically tractable animal model can additionally facilitate the subsequent unpacking of the mechanistic basis of different stages in perceptual dynamics. However, despite the recent rise in the use of the laboratory mouse for the study of the visual system (Huberman and Niell, 2011; Glickfeld et al., 2014; Seabrook et al., 2017) and of visually guided decision-making (Prusky et al., 2000; Prusky and Douglas, 2004; Busse et al., 2011; Histed et al., 2012; Carandini and Churchland, 2013; Glickfeld et al., 2013; Long et al., 2015; Burgess et al., 2017; Wang and Krauzlis, 2018; Speed et al., 2020; You and Mysore, 2020), the temporal dynamics of visual perceptual decisions represents a significant gap in mouse visual psychophysics (Histed and Maunsell, 2014; Umino et al., 2018; Nomura et al., 2019).

In this study, we adapted approaches from human psychophysical studies to investigate the dynamics of visual decision-making in freely behaving mice. In a series of experiments involving touchscreen-based (Mar et al., 2013; You and Mysore, 2020), two-alternative forced choice (2-AFC) orientation discrimination tasks, we investigated the effect of stimulus size, luminance, duration, delay, and the presence of a competing foil on mouse decision performance [accuracy and reaction time (RT)], and importantly, on the conditional accuracy function (CAF). We identified two distinct stages in the time course of mouse visual decision-making within a trial, as has been reported in humans (Posner and Keele, 1967; Phillips and Baddeley, 1971; Dick, 1974; Coltheart, 1980; Shibuya and Bundesen, 1988; Busey and Loftus, 1994; Vogel et al., 2006; Bays et al., 2011). In the first “sensory encoding” stage (Shibuya and Bundesen, 1988; Busey and Loftus, 1994; Vogel et al., 2006; Bays et al., 2011), response accuracy exhibited a classic trade-off with response speed, and asymptoted to a peak level. In the next stage, response accuracy did not exhibit such a trade-off, but instead, decayed following stimulus offset, consistent with a classic short-term memory (STM)-dependent process (Posner and Keele, 1967; Phillips and Baddeley, 1971; Dick, 1974; Coltheart, 1980). Combining these results with those from drift diffusion modeling (DDM; Ratcliff et al., 2016) allowed us to estimate fundamental psychophysical constants in mouse perceptual decision-making: the time needed by mice to complete visual sensory encoding, the duration for which their STM can intrinsically support discrimination behavior after stimulus input is removed, and the shortest visual stimulus duration that is informative. Additionally, by varying stimulus onset delay, we demonstrated that the two components of accuracy, namely, the CAF and the RT distribution can be independently modulated by task parameters. This also allowed a quantitative estimation of impulsivity of mice. Together, this study reveals parallels between mouse and human visual decision dynamics, despite differences in their sensory apparatuses, and enable investigations into the neural circuit underpinnings of the time course of perceptual decision-making in mice.

## Materials and Methods

### Animals

Thirty-seven mice (33 C57B16/J mice, all male; four PV-Cre mice, three female; The Jackson Laboratory) were housed in a temperature-controlled (∼75°F) and humidity-controlled (∼55%) facility on a 12/12 h light/dark cycle; zeitgeber time (ZT)0 = 7 A.M. All procedures followed the NIH guidelines and were approved by the Johns Hopkins University Animal Care and Use Committee (ACUC). Animals were allowed to acclimate for at least one week, with *ad libitum* access to food and water before water regulation was initiated per previously published procedures (Guo et al., 2014). Briefly, mice were individually housed (for monitoring and control of daily water intake of each identified animal), and administered 1 ml of water per day to taper their body weight down, over the course of 5–7 d, to 80–85% of each animal’s free-feeding baseline weight. During behavioral training/testing, the primary source of water for mice was as a reinforcer for correct performance: 10 μl of water was provided for every correct response. Experiments were all conducted in the light phase.

### Apparatus

**Figure 1.**
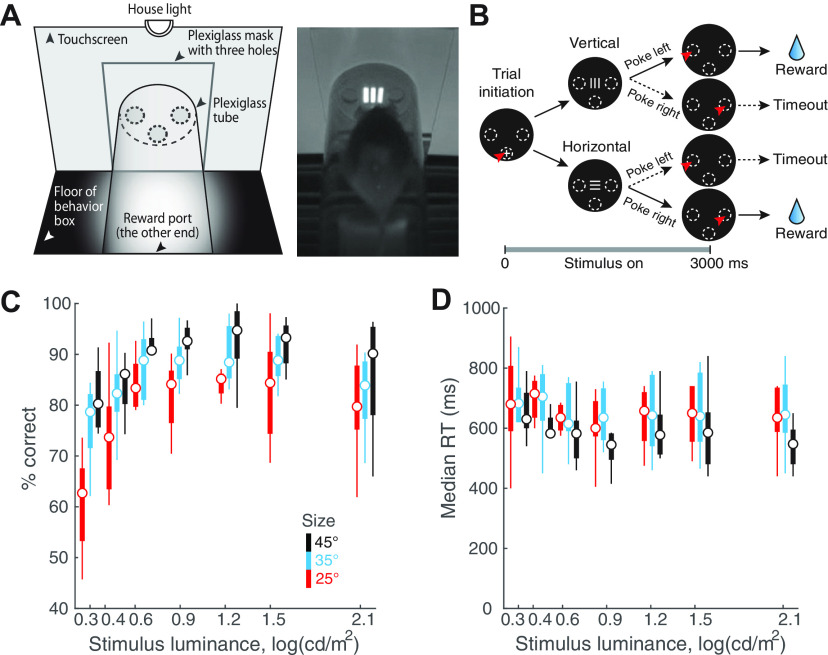
Stimulus luminance and size modulate orientation discrimination performance in freely behaving mice. ***A***, Left, Schematic of touchscreen-based experimental setup showing key components. Right, Snapshot of freely behaving mouse facing a visual stimulus on the touchscreen. ***B***, Schematic of 2-AFC task design. Black discs, Screenshots of touchscreen with visual stimuli. Dashed ovals, Locations of holes through which mice can interact with touchscreen; white “+”: zeroing cross presented within central response hole at start of each trial. Red arrowhead, Nose-touch by mouse. Shown also are vertical or horizontal grating stimuli, and reinforcement (water)/punishment (timeout) schedule. Bottom, Trial timeline. 0 ms corresponds to the instant at which the mouse touches the zeroing cross (trial initiation). Immediately following this, the target grating was presented and stayed on for 3 s, or until the mouse responded, whichever came first. Vertical and horizontal targets were interleaved randomly. ***C***, Psychometric plots of discrimination accuracy against stimulus luminance (*n* = 8 mice). Different colors correspond to different target sizes. Two-way ANOVA, *p* < 0.001 (luminance), *p* < 0.001 (size), *p* = 0.498 (interaction). Effect size η^2^ = 0.292 (luminance), η^2^ = 0.192 (size), η^2^ = 0.037 (interaction). For each stimulus size/luminance, the box plot shows the median (the central mark) and the 25th and 75th percentiles (the bottom and top edge of the box) of the group (*n* = 8). The whiskers extend to the most extreme data points not considered as outliers. ***D***, Plot of median RT against stimulus luminance. Two-way ANOVA, *p* = 0.998 (luminance), *p* = 0.004 (size), *p* = 1 (interaction). Effect size η^2^ = 0.003 (luminance), η^2^ = 0.071 (size), η^2^ = 0.010 (interaction).

Behavioral training and testing were performed in soundproof operant chambers equipped with a touchscreen (Med Associates Inc.), a custom-built reward port (fluid well), infrared video cameras, a house light and a magazine light above the reward port. The reward port was located at the opposite wall of the chamber relative to the touchscreen (Fig. 1*A*; Extended Data Fig. 1-1*A*). Mice were placed within a clear Plexiglas tube (5 cm in diameter) that connects the touchscreen and the reward port. A thin Plexiglas mask (3-mm thickness) was placed 3 mm in front of the touchscreen with three apertures (1 cm in diameter) through which mouse was allowed to interact with the screen via nose-touch. The “left” and “right” apertures were placed 3 cm apart (center-to-center) along the base of the triangle, and a “central” aperture, at the apex of the triangle, was 1.5 cm below the midpoint of the base. All experimental procedures were executed using control software (K-limbic, Med Associates).

10.1523/ENEURO.0161-21.2022.f1-1 Extended Data Figure 1-1 Extended data for Figure 1. ***A***, Lateral view of the schematic experimental setup showing the relative position of the touchscreen (leftmost vertical line), the plexiglass mask (grey-filled vertical bar), and the tube within which mice move (50 mm in diameter); the plexiglass mask is positioned 3 mm in front of the touchscreen. Dashed lines indicate the central response hole (lower dashed lines), and left/right response holes (upper dashed lines; 10 mm in diameter). For single-stimulus discrimination, the center of the stimulus is aligned with the center of left/right response holes in elevation, and with the central hole in azimuth (see Fig. 1*A*). For experiments involving two stimulus locations (i.e., flanker task), the upper (magenta) and lower (cyan) locations of the stimulus are indicated as colored bars (see also Fig. 4*A*). The 60 × 60 pixels (12 × 12 mm) stimulus subtends a visual angle of 25° when viewed from 20 mm front of the plexiglass mask. ***B***, Identification of trials towards the end of the 30-min behavioral sessions that corresponded to animals being poorly engaged in the task (Materials and Methods; You and Mysore, 2020). Top panel, Time course of overall response accuracy across mice as a function of trial number within sessions. Accuracy obtained from trials pooled across all mice and sessions, and computed as a function of trial number within session (blue; Materials and Methods). Grey shading, Bootstrapped estimates of the 95% confidence interval of the accuracy (gray; Materials and Methods). Diamonds on top, Trials whose accuracy not significantly different from chance. Dashed vertical line, First trial at which the accuracy was not different from chance (50%), and stayed indistinguishable from chance for 3/5 of the next five trials (Materials and Methods). Data show increased variability and worse performance towards the end of sessions. Bottom panel, Number of actual observations across mice for each trial number, as a percentage of the maximal number of possible observations (Σ mice × sessions), plotted as a function of trial number within session (red). Solid vertical line, First trial at which the number of observations drops below 25%. Data show drop in the number of observations available to reliably assess performance towards the end of sessions. Based on these data, all trials above 122 of each behavioral session of this experiment were dropped from analysis (Materials and Methods). Results in Figure 1 are based on data from trials 1–122 from each behavioral session. ***C***, Response accuracy as a function of stimulus size (*n* = 9 mice; *p* = 0.001, one-way ANOVA). In these experiments, stimulus size was manipulated independently (without manipulation of luminance; unlike in Fig. 1). All stimuli were at the highest luminance (130 cd/m^2^). ***D***, Median RT as a function of stimulus size (*n* = 9 mice; *p* = 0.205, one-way ANOVA). Download Figure 1-1, EPS file.

### Visual stimuli

Visual stimuli were bright objects on the dark background (luminance = 1.32 cd/m^2^). A small cross (60 × 60 pixels; luminance = 130 cd/m^2^) was presented in the central aperture and had to be touched to initiate each trial. Oriented gratings (horizontal or vertical) were generated using a square wave, with fixed spatial frequency (24 pixel/cycle, ∼0.1 cycle/degree from 2cm viewing distance, Extended Data Fig. 1-1*A*) known to be effective for mice to discriminate (Histed et al., 2012). The dark phase of the grating was black, identical to the background (luminance, L_dark_= 1.32 cd/m^2^), and the bright phase was varied between 1.73 and 130 cd/m^2^ depending on the tasks (see below). Note that as the luminance of the bright phase of the grating changed, the contrast of the grating also changed. For clarity, we refer to this stimulus manipulation as a change in luminance, throughout. The size of the stimulus was also varied depending on the task, ranging from 60 × 60 to 108 × 108 pixels, which subtended 25–45 visual degrees at a viewing distance of 2 cm from the screen (Extended Data Fig. 1-1*A*).

### Experimental procedure and behavioral training

Each mouse was run for one 30-min behavioral session per day, with each session yielding 80–180 trials. Each trial in a session was initiated by the mouse touching the zeroing cross. Upon trial initiation, the cross vanished, and the visual stimulus (or stimuli) were immediately presented (except in the delay task), for a duration of 0.1–3 s depending on the task (see below). Mice were trained to report the orientation of target grating, by nose-touching the correct response aperture (vertical → left; horizontal → right). A correct response triggered a tone (600 Hz, 1 s), the magazine light turning on, and the delivery of 10 μl of water. When mice turned to consumed the reward, their head entry into the reward port was detected by an infrared sensor which caused the zeroing cross (for the next trial) to be presented again. An incorrect response triggered a 5-s timeout, during which the house light and the magazine light were both on and zeroing cross was unavailable for the next trial to be initiated. A failure to respond within 3 s (starting stimulus presentation) resulted in a trial reset: the stimulus vanished and the zeroing cross was presented immediately (without a timeout penalty), to allow initiation of the next trial. Well-trained animals failed to respond on fewer than 5% of the total number of trials, and there were no systematic differences in the proportion of such missed trials between different conditions. Within each daily 30-min behavioral session, mice consumed ∼1 ml of water. If a mouse failed to collect enough water from the behavioral session, they were provided with a water supplement using a small plastic dish in their home cage.

### Single-stimulus discrimination task

**Figure 2.**
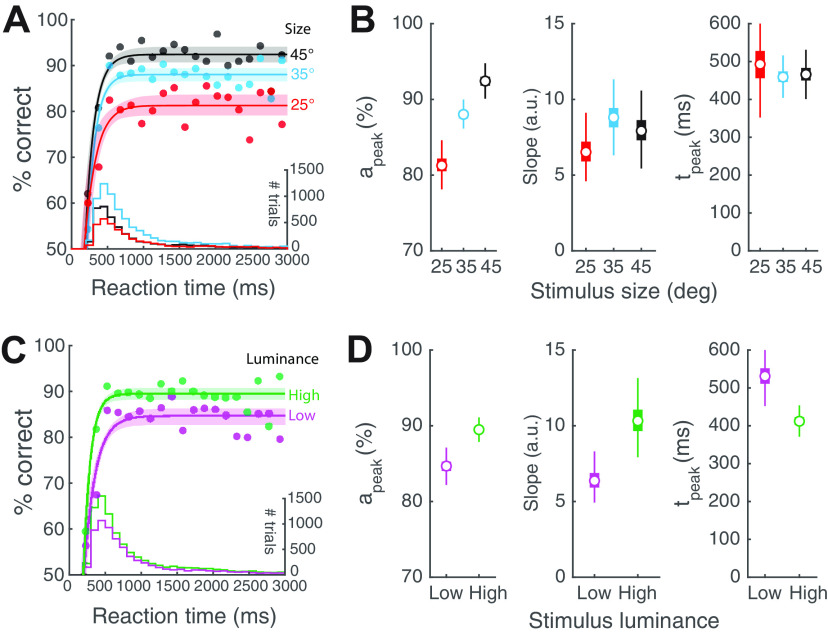
Stimulus size and luminance modulate the sensory encoding stage of the CAF. ***A***, Plot of accuracy as a function of RT bins (conditional accuracy) using same dataset as Figure 1. Data pooled across all stimulus luminance and mice (*n* = 8), sorted by stimulus size; RT bin size = 100 ms. Solid curves, CAFs (best-fit rising asymptotic function; Materials and Methods) for targets of different sizes (black: 45°; blue: 35°; red: 25°). Light shading, 95% confidence interval of the fit (abbreviated as C.I., henceforth; Materials and Methods). Histograms at bottom, RT distributions for targets of different sizes (*y*-axis on the right). The overall response accuracy for a particular stimulus condition is the dot product of the CAF and the RT distribution. ***B***, Box plots of the key parameters for different target sizes. Left panel, a_peak_. Middle panel, Slope parameter. Right panel, t_peak_. ***C***, CAFs for targets of different luminance conditions (magenta: “low” luminance, first three luminance levels from Fig. 1*C*; green: “high” luminance, last four luminance levels; Materials and Methods); conventions as in ***A***. ***D***, Box plots of the key parameters for different luminance conditions; conventions as in ***B***. The box plots in all panels show the median (open circle), the 25th and 75th percentiles (the bottom and top edge of the box), and the most extreme data points not considered as outliers (whiskers); in some panels, the boxes are the same size as the symbol for the median.

**Figure 3.**
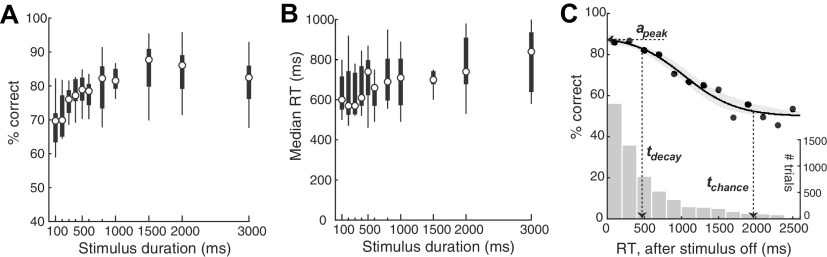
Stimulus duration and the memory-dependent stage of the CAF. ***A***, Psychometric plot of discrimination accuracy against stimulus duration (*n* = 9 mice; one-way ANOVA; *p* < 0.001; effect size η^2^ = 0.331). ***B***, Plot of median RT against stimulus duration (one-way ANOVA; *p* = 0.056; effect size η^2^ = 0.177). ***C***, Plot of the conditional accuracy (solid data) as a function of RT bins relative to stimulus offset. Only trials in which the stimulus was longer than 332 ms were included (to ensure full sensory encoding, see text; Materials and Methods). Curve and shading, Best-fit sigmoid function and 95% C.I. Bootstrapped estimates of each key metric: a_peak_, median [C.I.] = 87.3 [84.8, 89.9] %; t_decay_ = 469 [279, 697] ms; and t_chance_ = 1969 [1708, 2520] ms. Histogram, RT distribution (*y*-axis on the right). In this experiment, stimulus size and luminance were maintained fixed at 25° and 130 cd/m^2^, respectively.

Upon trial initiation, a single grating stimulus (i.e., the “target”) was presented above the central aperture, at the same horizontal level as the left and right apertures, and mice were required to report its orientation with the appropriate nose-touch (Fig. 1*B*). When stimulus size and luminance were manipulated (Figs. 1, 2), three different sizes were tested: 60 × 60, 84 × 84, 108 × 108 (pixels × pixels). For each size, seven different levels of luminance were tested: 2.00, 2.59, 4.37, 7.55, 16.2, 34.3, 130 cd/m^2^. [These corresponded nominally to Michelson’s contrasts of 20%, 32%, 54%, 70%, 85%, 93%, 98%, respectively; Michelson’s contrast is computed as (luminance_bright_ – luminance_dark_)/(luminance_bright_ + luminance_dark_) × 100.] Trials with different stimulus luminance at a particular size were interleaved randomly throughout a session, while trials with different stimulus sizes were examined on different days. When the stimulus duration was manipulated (Fig. 3), the luminance (130 cd/m^2^) and size (60 × 60 pixels) of the grating were fixed, and eleven different stimulus durations were tested: 100, 200, 300, 400, 500, 600, 800, 1000, 1500, 2000, 3000 ms. The stimulus duration was fixed for a given day, and across days, was varied in a descending sequence from 3000 to 100 ms. When the stimulus onset delay was manipulated, the luminance (130 cd/m^2^), size (60 × 60 pixels), and duration (600 ms) of the grating were fixed. Three different delays were tested: 0, 100, and 200 ms. The delay duration was fixed for a given day, and varied in an ascending sequence from 0 to 200 ms.

10.1523/ENEURO.0161-21.2022.f2-1 Extended Data Figure 2-1 Extended data for Figure 2. CAFs of individual mice and effect of bin size. ***A***, The general pattern of conditional accuracy curves across mice. Each color represents one single mouse. Each curve was generated by pooling all trials (of various stimulus size and luminance) from one mouse, sort the trials by RT, and then do a moving average (window size = 200 trials) to plot the mean accuracy (y) at mean RT (x) of the time window. ***B–D***, Fitting of the CAF in various bin sizes. ***B***, Bin size = 30 ms. ***C***, Bin size = 60 ms. ***D***, Bin size = 150 ms. ***E–G***, Estimates of the quantitative metrics of the CAF in various bin sizes. ***E***, Peak conditional accuracy (*a_peak_*). ***F***, Slope parameter. ***G***, Time to reach peak conditional accuracy (t_peak_). Download Figure 2-1, EPS file.

10.1523/ENEURO.0161-21.2022.f2-2 Extended Data Figure 2-2 Extended data for Figure 2. Estimates of all four parameters of the drift diffusion model. ***A***, Drift rate; two-way ANOVA, *p* = 0.028 (luminance), *p* < 0.001 (size), *p* = 0.767 (interaction). ***B***, Boundary separation; two-way ANOVA, *p* = 0.171 (luminance), *p* = 0.026 (size), *p* = 0.953 (interaction). ***C***, Starting point; two-way ANOVA, *p* < 0.001 (luminance), *p* = 0.325 (size), *p* = 0.098 (interaction). ***D***, t_delay_; two-way ANOVA, *p* = 0.523 (luminance), *p* = 0.308 (size), *p* = 0.931 (interaction). Download Figure 2-2, EPS file.

10.1523/ENEURO.0161-21.2022.f3-1 Extended Data Figure 3-1 Extended data for Figure 3. CAFs of individual mice and effect of bin size. ***A***, Identification of trials towards the end of the 30-min behavioral sessions that corresponded to animals being poorly engaged in the task (Materials and Methods); conventions identical to those in Extended Data Figure 1-1*B*. ***B***, The general pattern of conditional accuracy curves across mice. Each color represents one single mouse. Each curve was generated by pooling all trials (of various stimulus size and luminance) from one mouse, sort the trials by RT, and then do a moving average (window size = 200 trials) to plot the mean accuracy (y) at mean RT (x) of the time window. ***C–E***, Fitting of the CAF in various bin sizes. ***C***, Bin size = 50 ms. ***D***, Bin size = 130 ms. ***E***, Bin size = 260 ms. ***F–H***, Estimates of the quantitative metrics of the CAF in various bin sizes. ***F***, Peak conditional accuracy (*a_peak_*). ***G***, The time at which conditional accuracy started to decay (t_decay_). ***G***, The time at which conditional accuracy fell to the chance level (t_chance_). Download Figure 3-1, EPS file.

### Flanker task

**Figure 4.**
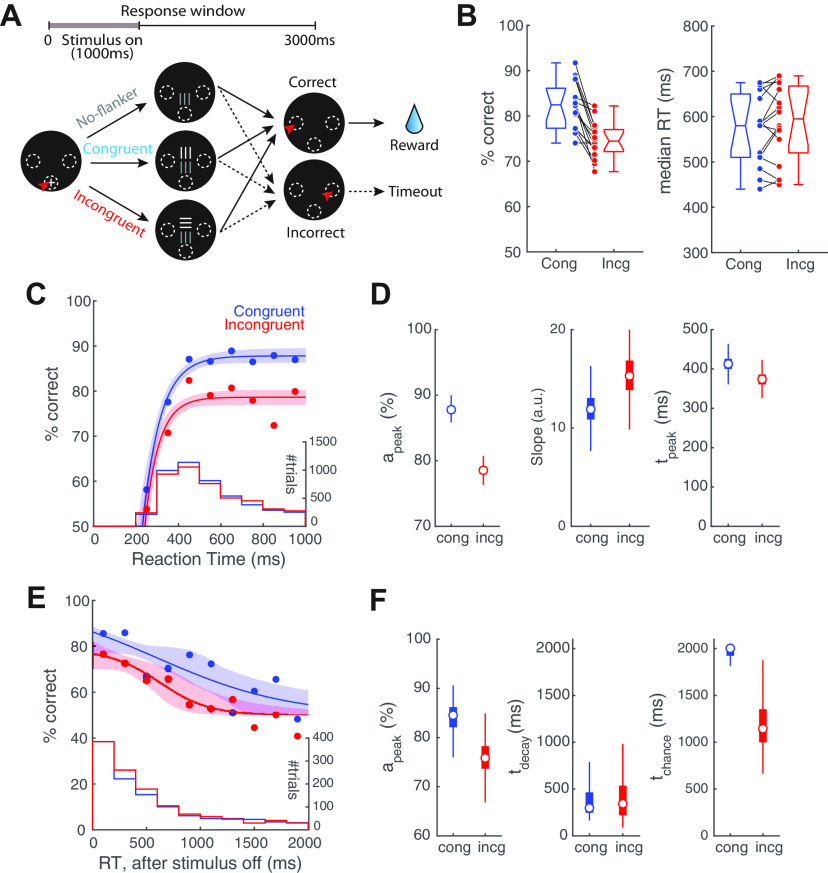
Incongruent flanker modulates the sensory encoding stage of the CAF. ***A***, Schematic of the flanker task; target grating is always presented at the lower location; a second “flanker” grating (orthogonal orientation, incongruent flanker; or same orientation, congruent flanker) is presented simultaneously, and always at the upper location; luminance of flanker is systematically varied (adapted from You and Mysore, 2020). All other conventions as in Figure 1. The stimuli were presented for 1 s, and the response window was 3 s. ***B***, Left panel, Comparison of performance between trials with incongruent versus congruent flanker. *p* < 0.001, paired-sample *t* test. Effect size Hedges’ g = 1.61. Right panel, Comparison of median RT between trials with incongruent versus congruent flanker. *p* = 0.137, paired-sample *t* test. Effect size Hedges’ g = −0.176. Data re-analyzed from You and Mysore (2020); each line represents data from one mouse (*n* = 17 mice). Data in ***B–F*** include only trials with high flanker luminance (≥20.1 cd/m^2^; see text). ***C***, CAFs of the sensory encoding stage. Blue, Trials with congruent flanker. Red, Trials with incongruent flanker. Histograms, RT distributions. ***D***, Key parameters of CAFs (sensory encoding stage) for trials with congruent versus incongruent flanker; a_peak_ (left), slope parameter (middle), and t_peak_ (right). Box plots show the distribution of bootstrapped estimates (Materials and Methods). Effect sizes (congruent–incongruent): a_peak_: Hedges’ g = 11.0; slope parameter: Hedges’ g = −1.73; t_peak_: Hedges’ g = 2.08. Note, the sizes of the boxes in the left and right panels are similar to the sizes of the circular symbols depicting the medians. ***E***, CAFs of the STM-dependent stage; data aligned to stimulus offset. Blue, Trials with congruent flanker. Red, Trials with incongruent flanker. ***F***, Plots of key parameters of CAFs (STM-dependent stage) for trials with congruent versus incongruent flanker; a_peak_ (left), t_decay_ (middle), and t_chance_ (right). Conventions and statistical methods as in ***D***. a_peak_: Hedges’ g = 2.54; t_decay_: Hedges’ g = 0.175; t_chance_: Hedges’ g = 2.98.

Upon trial initiation, either one stimulus (“target,” 60 × 60 pixels, luminance = 20.1 cd/m^2^) was presented at the lower location, or two stimuli were presented simultaneously, with the target at the lower location and a second “flanker” at the upper location (Fig. 4*A*). Flankers were of the same size (60 × 60 pixels) and spatial frequency (24 pixels/cycle) as the target, but with luminance ranging (over eight levels) from less than that of the target to greater than that of the target (You and Mysore, 2020). The orientation of the flanker was either identical to that of the target (“congruent trial”) or orthogonal to that of the target (“incongruent trial”). The stimulus (stimuli) was (were) presented for a duration of 1 s, and mice were required to report orientation of the target grating with the appropriate nose-touch (within 3 s). All types of trials (no flanker, congruent, incongruent) and flanker luminance were interleaved randomly within each daily session. Data from this experiment have been reported previously (You and Mysore, 2020), and were re-analyzed here using different analyses.

### Subject inclusion/exclusion

A total of 37 mice were used in this study, with different subsets used in different tasks. For mice involved in more than one task, they were well rested for three to eight weeks with food and water *ad libitum* between experiments. Before the start of each experiment, all mice were given a few days of practice session to ensure that they remembered/re-learned the association between the orientation of single target and the appropriate nose-touch. Of the total of 37 mice trained across tasks, 28 mice passed the inclusion threshold of response accuracy >70% in the single-stimulus discrimination task, and were included for the analyses reported in this paper.

### Trial inclusion/exclusion

Mice were observed to become less engaged in the task toward the end of a behavioral session, when they had received a sizeable proportion of their daily water intake. This was reflected in their behavioral metrics: they tended to wait longer to initiate the next trial, and their performance deteriorated. We identified and excluded such trials following a published procedure (You and Mysore, 2020), to minimize confounds arising from loss of motivation toward the end of sessions. Briefly, we pooled data across all mice and all sessions, treating them as coming from one session of a single “mouse.” We then binned the data by trial number within the session, computed the discrimination accuracy in each bin (% correct), and plotted it as a function of trial number within session (Extended Data Figs. 1-1*B*, 3-1*A*, 5-1*A*). Using a bootstrapping approach, we computed the 95% confidence interval for this value. We used the following exclusion criterion: trials q and above were dropped if the q^th^ trial was the first trial at which at least one of the following two conditions was satisfied: (1) the performance was statistically indistinguishable from chance on the q^th^ trial and for the majority (3/5) of the next five trials (including the q^th^); (2) the number of observations in q^th^ trial was below 25% of the maximum possible number of observations for each trial (Σ mice × sessions), thereby signaling substantially reduced statistical power available to reliably compare performance to chance. The plots of performance as a function of trial number, and number of observations as a function of trial number for the different tasks in this study are shown in Extended Data Figures 1-1*B*, 3-1*A*, 5-1*A*, along with the identified cutoff trial numbers (q).

### Behavioral measurements

**Figure 5.**
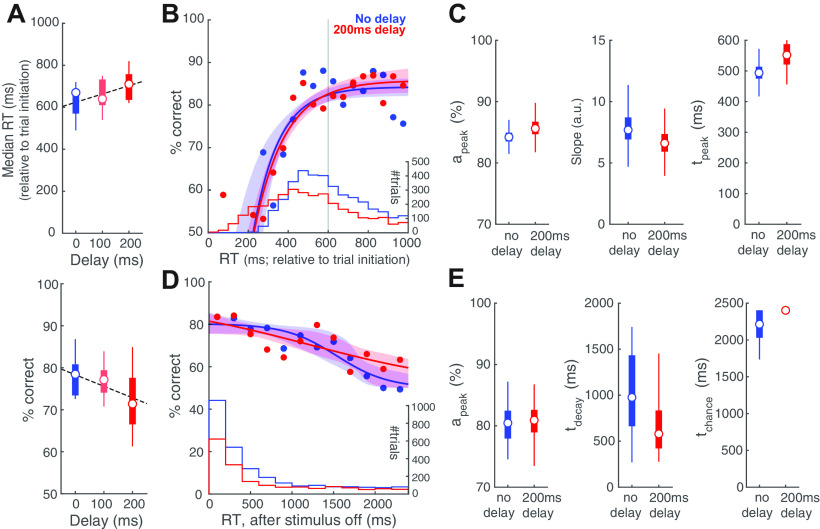
Stimulus onset delay modulates RT distribution but not the CAF. ***A***, Upper, Plot of median RT, measured relative to trial initiation, against stimulus onset delay (*n* = 9 mice; *p* = 0.094, one-way ANOVA; effect size η^2^ = 0.179; Pearson’s correlation = 0.422, *p* = 0.028). Dashed line, Linear regression on RTs. Lower, Plot of response accuracy against stimulus onset delay (*p* = 0.182, one-way ANOVA; effect size η^2^ = 0.132; Pearson’s correlation = −0.358, *p* = 0.067). ***B***, CAFs of the sensory encoding stage. Blue, Trials with no delay. Red, Trials with 200-ms delay. Shaded bands, Bootstrapped confidence intervals (95%); confidence intervals overlap for the two datasets. Histograms, RT distributions. Gray vertical line, Stimulus offset. ***C***, Key parameters of the CAF (sensory encoding stage) for trials with no delay versus trials with 200-ms delay. Box plots show the distribution of the bootstrapped estimates. ***D***, CAFs of the STM-dependent stage. Conventions as in ***B***. ***E***, Key parameters of the CAF (STM-dependent stage) for trials with no delay versus trials with 200-ms delay. Conventions as in ***C***.

Response accuracy (% correct) was calculated as the number of correct trials divided by the total number of trials responded (correct plus incorrect). RT was defined as the time between the start of stimulus presentation and time of response nose-touch, both detected by the touchscreen. In the experiment involving stimulus onset delays (Fig. 5*A*), RT was computed with respect to trial initiation (as opposed to from stimulus onset).

10.1523/ENEURO.0161-21.2022.f5-1 Extended Data Figure 5-1 Extended data for stimulus onset delay experiment. Identification of trials towards the end of the 30-min behavioral sessions that corresponded to animals being poorly engaged in the task (Materials and Methods). All conventions are as in Extended Data Figure 1-1*B*. Based on these data, all trials above 116 of each behavioral session of this experiment were dropped from analysis. Results in Figure 5 are based on data from trials 1–116 from each behavioral session. Download Figure 5-1, EPS file.

### DDM of RT distributions

The RT measured here represents the duration from stimulus onset to completion of the motor response. In order to specifically isolate the time spent in decision-making (separately from the latency of activation of sensory neurons as well as duration of motor execution), we applied the drift-diffusion model to our RT data (Voss et al., 2013, 2015). This model hypothesizes that a subject (“decision-maker”) collects information from the sensory stimulus via sequential sampling, causing sensory evidence to accrue for or against a particular option (usually binary) while viewing the stimulus. A decision is to be made when the accumulating evidence reaches an internal threshold of the subject. This process of evidence accumulation, together with the processes of sensory encoding and motor execution, as well as threshold crossing, determine the RT observed on each trial.

We used a standard version of the model that consists of four independent variables (Ratcliff, 1978; Ratcliff et al., 2016): (1) the drift rate; (2) the boundary separation; (3) the starting point; and a (4) nondecisional constant (t_delay_), which accounts for the time spent in sensory transmission and motor execution. In the case of our tasks, there was no reason for the drift rate to be different between vertical versus horizontal gratings, and therefore, we merged both type of trials (trials with a horizontal target grating and trials with a vertical target grating). We treated “correct” response and “incorrect” response as the two binary options, and fit the diffusion model to the RT distributions of correct versus incorrect trials using the fast-dm-30 toolbox with the maximum likelihood option to gain estimates of those four parameters for each individual mouse (Extended Data Fig. 2-2; Voss et al., 2015).

### Conditional accuracy analysis

Conditional accuracy was calculated as the percentage of correct trials (accuracy) as a function of RT. For this analysis, trials from all mice were pooled together and treated as if they were from one single mouse for statistical power (Fig. 2 onwards; for completeness, conditional accuracy plots using nonpooled data, i.e., from individual mice, are included in Extended Data Figs. 2-1*A*, 3-1*B*). Pooled trials were then sorted by their RT, and then binned by RT such that there were: (1) sufficient number of trials in each bin; and (2) sufficient number of total bins, to ensure the robustness of curve fitting and therefore the estimates of quantitative metrics (see below). Typical bin sizes used were 50-, 100-, or 200-ms bins, depending on the experiments and stage of analysis (sensory encoding or STM-dependent). The effect of bin size on the estimates of quantitative metrics is explored in the Extended Data Figures 2-1*B-G*, 3-1*C-H*; results show that the estimates are comparable across tested bin sizes.

### CAF

To quantitatively describe the relationship between the conditional accuracy and RT, we fitted the plot of accuracy against binned RT with parametric functions (the CAF; see below) using a nonlinear least square method. For RT bins aligned to stimulus onset (Figs. 2, 4*C*, 5*B*), we fit the conditional accuracy data using an increasing asymptotic function:

$$\text{Conditional accuracy }=\lambda \left(1\;-{\;\text{e}}^{-\gamma_{enc}(\text{RT}-\delta )}\right).$$

Three key metrics were defined for this sensory encoding phase, for use in subsequent comparisons across conditions: (1) peak conditional accuracy (a_peak_), the maximal level of accuracy that the CAF reaches within the range of RT; (2) the slope parameter (*γ_enc_*); and (3) the first instant at which the conditional accuracy reaches its maximal value (t_peak_), defined as the time point at which the ascending CAF reaches 95% of a_peak_. Note that t_peak_ is influenced by the peak conditional accuracy (a_peak_), the slope parameter, *γ_enc_*, and the temporal offset at chance performance, δ. For RT bins aligned to stimulus offset (Figs. 3*C*, 4*E*, 5*D*), we fit the decaying conditional accuracy data using a sigmoidal function:

$$\text{Conditional accuracy }=\lambda [\text{1}/(\text{1}\;+{\;\text{e}}^{-}{{}^{{\text{β}}_{\text{dec}}\;(\text{RT}-}}^{\tau )})]+\text{5}0.$$

Three key metrics were defined for this STM-dependent stage for use in subsequent comparisons across conditions: (1) peak conditional accuracy (a_peak_), the maximal level of accuracy within the range of RT; (2) the first instant (t_decay_) at which conditional accuracy is lower than the maximum, defined as the time point at which the descending CAF crosses 95% of a_peak_; and (3) the first instant (t_chance_) at which conditional accuracy drops to chance level, defined as the time point at which the descending CAF crosses 52.5%. In (rare) cases when the CAF never went below 52.5%, t_chance_ was set to be the upper bound of the window of analysis (i.e., 3000 ms – stimulus duration = the window for which the mice can respond following stimulus offset). Note that t_decay_ and t_chance_ are influenced by both the slope parameter, β_dec_, and τ.

Confidence intervals of the CAF fits, as well as for the parameters, were estimated by standard bootstrapping procedures involving resampling the raw data randomly with replacement (1000×), to get repeated estimates of the CAF and corresponding metrics. In all relevant figures, the box plots of the estimated values of each metric show the median (the central mark), the 25th and 75th percentiles (the bottom and top edge of the box), and the most extreme data points not considered as outliers (whiskers).

In the experiment in which the stimulus onset delay was manipulated (Fig. 5), we adopted the following two adjustments to our procedure for the analysis of the CAF. First, since the stimulus was short (600 ms), to ensure robust estimates of CAF metrics for the sensory encoding stage, we included data beyond stimulus offset as well for the fitting of the CAF through 400 ms following offset. (We chose to include data up to 400 ms after offset, specifically, because we had learned from Fig. 3 that conditional accuracy remains at its plateau for nearly 500 ms following stimulus offset.) Second, we also excluded trials with RT <200 ms for the fitting of the CAF (Fig. 5*B*), because these represent trials on which responses were initiated prematurely, before the stimulus was even presented (200 ms represents our estimate of the duration of sensory latency plus motor execution; see text to Fig. 2).

### Statistical tests

All analyses and statistical tests were performed in MATLAB. For single-stimulus experiments in which only one stimulus parameter was systemically varied, one-way ANOVA was applied to examine the effect of the manipulating the single factor (duration and delay; Figs. 3*A*,*B*, 5*A*; Extended Data Fig. 1-1*C*,*D*). For experiments that involved changing both stimulus size and luminance (Fig. 1*C,D*; Extended Data Fig. 2-2), two-way ANOVA was applied to examine the effect of each factor, as well as their interaction. For the flanker task, the paired-sample *t* test was used to examine whether the group performance was different between trial types (Fig. 4*B*).

For the metrics associated with CAF, comparisons were made by measuring the effect size (Hedges’ g) of the difference between two distributions (Figs. 2*B*,*D*, 4*D*,*F*, 5*C*,*E*). All effect size measurements, including those with ANOVA (η^2^), were calculated following the methods (and source code) of Hentschke and Stüttgen (2011). Hedges’ g estimates the distance between the two distributions in units of their pooled standard deviation, with larger numbers indicating stronger effects. η^2^ varies from 0 to 1, with larger values indicating greater ratio of variance explained in the dependent variable by a predictor while controlling the other variables.

## Results

In this study, freely behaving mice were trained to perform 2-AFC orientation discrimination in a touchscreen-based setup (Mar et al., 2013; You and Mysore, 2020; Materials and Methods). Briefly, mice were placed in a Plexiglas tube within a soundproof operant chamber equipped with a touch-sensitive screen at one wall and a reward well at the opposite wall (Fig. 1*A*). A Plexiglas sheet with three holes was placed in front of the touchscreen, the holes corresponded to the locations at which mice were allowed to interact with the screen by a nose-touch (Fig. 1*A*). All trials began with a nose-touch on a bright zeroing-cross presented within the lower central hole (Fig. 1*B*). Immediately following nose-touch, an oriented grating (target; bright stimulus on a dark background) was presented at the center of the screen. Mice were rewarded if they responded to the orientation of the target with an appropriate nose-touch: vertical (horizontal) grating → touch within upper left (upper right) hole. Behavioral data were collected from daily sessions that lasted 30 min for each mouse (see also Extended Data Fig. 1-1).

### Stimulus size and luminance modulate mouse discrimination performance

We first examined the effect of target size and luminance on the decision performance of mice in the orientation discrimination task. Here, the target grating was presented for up to 3 s after trial initiation (Fig. 1*B*; Materials and Methods), and its size and luminance were systematically varied; the spatial frequency was fixed at 0.1 cycles/degree (24 pixels/cycle; Prusky and Douglas, 2004; Histed et al., 2012; Materials and Methods). Mice were allowed to respond at any time during stimulus presentation, and the stimulus was terminated automatically on response.

We found that both the target luminance and size significantly modulated discrimination accuracy (two-way ANOVA, main effect of luminance, *p* < 0.001, effect size η^2^ = 0.292; main effect of size, *p* < 0.001, η^2^ = 0.192; interaction, *p* = 0.498, η^2^ = 0.037; Fig. 1*C*). These results revealed that mice discriminated target orientation better than chance even at the lowest luminance (2.00 cd/m^2^) and size (25°) tested (the red box at the left lower corner, *p* = 0.015, *t* test against mean accuracy = 50%, effect size g1 = 1.129; Fig. 1*C*). Additionally, at this smallest target size (25^0^), mice could discriminate with >80% accuracy for most of the tested luminance values (≥4.37 cd/m^2^; Fig. 1*C*, red data).

The effect of these parameters on median RTs was less pronounced. Target size, but not luminance, modulated RTs (two-way ANOVA; main effect of size, *p* = 0.004, effect size η^2^ = 0.071; main effect of luminance, *p* = 0.998, η^2^ = 0.003; interaction, *p* = 1, η^2^ = 0.010; Fig. 1*D*). Together, these results revealed a systematic effect of target size and luminance on discrimination accuracy.

### Effect of stimulus size and luminance on dynamics of visual decision-making: the sensory encoding stage

To investigate the dynamics of visual perceptual decision-making, we adapted approaches from human studies and examined the dependence of response accuracy on RT, i.e., the so-called CAF (Wickelgren, 1977; McElree and Dosher, 1989; Heitz, 2014). For these analyses, we pooled trials from all mice (*n* = 8) to gain better statistical power for the estimates of parameters of the CAF (Materials and Methods; plots of the data for individual mice showed similar overall shapes of the CAF; Extended Data Fig. 2-1*A*).

Specifically, we investigated the dynamics of visual perceptual decision-making as a function of stimulus size, and separately, as a function of stimulus luminance. First, to examine the effect of stimulus size on decision dynamics, we pooled trials from all mice across luminance values (7 luminance values) for each stimulus size, sorted them based on RT, and plotted conditional accuracy for each RT bin (100 ms; Fig. 2*A*; Materials and Methods). We found that for responses with RT less than ∼500 ms, conditional accuracy improved for longer RT, consistent with the classic “speed-accuracy trade-off” (Heitz, 2014). For responses with RT >500 ms and up to 3 s, the allowed duration for responses, conditional accuracy plateaued, and was independent of RT. Next, to examine the effect of stimulus luminance on decision dynamics, we pooled trials from all mice across size values into two groups based on stimulus luminance: (1) trials with target luminance ≤4.37 cd/m^2^ (“low luminance”); and (2) trials with target luminance >4.37 cd/m^2^ (“high luminance”; Materials and Methods). Here, as well, we found a similar initial stage of increasing conditional accuracy up to RT of ∼500 ms, followed by a plateauing of conditional accuracy.

Drawing on arguments from human behavioral studies, we reasoned that the initial transient stage of the CAF reflects the process of sensory encoding: during it, slower responses allow more sensory evidence to be acquired, thereby improving conditional accuracy up to a peak value reflecting the completion of sensory encoding (Shibuya and Bundesen, 1988; Busey and Loftus, 1994; Vogel et al., 2006; Bays et al., 2011).

To quantify these dynamics, we fit the conditional accuracy data with an asymptotic function (Fig. 2*A*,*C*, solid curves; Wickelgren, 1977; McElree and Dosher, 1989; Heitz, 2014), and estimated three key metrics, in each case: (1) the peak conditional accuracy (a_peak_); (2) the slope parameter (γ_*enc*_); and (3) the time point at which conditional accuracy reached its peak (t_peak_; Materials and Methods).

We found that the peak conditional accuracy was significantly modulated by stimulus size (Fig. 2*B*, left; a_peak_: size 25°, median [95% confidence Interval, C.I.] = 81.3 [79.1, 83.7] %; size 35^0^ = 88.0 [86.5, 89.4] %; size 45^0^ = 92.4 [90.7, 94.1] %; effect size Hedges’ g = −6.71 (25° versus 35°), −5.39 (35° versus 45°), −10.6 (25° versus 45°)), but not the slope of the function (slope parameter, γ_*enc*_, Fig. 2*B*, middle; size 25° = 6.52 [5.10, 9.07] a.u.; size 35° = 8.81 [7.09, 10.6] a.u.; size 45° = 7.92 [6.15, 10.1] a.u.; Hedges’ g = −2.24 (25° versus 35°), 0.863 (35° versus 45°), −1.34 (25° versus 45°)), or the time to reach peak accuracy (t_peak_, Fig. 2*B*, right; size 25° = 493 [375, 597] ms; size 35° = 459 [420, 505] ms, size 45° = 466 [420, 522] ms; Hedges’ g = 0.728 (25° versus 35°), −0.274 (35° versus 45°), 0.558 (25° versus 45°)).

Next, we found that the peak conditional accuracy was higher in high-luminance trials (Fig. 2*D*, left; low-luminance, median [C.I.] = 84.7 [82.7, 86.3] %; high-luminance = 89.5 [88.2, 90.7] %, effect size Hedges’ g = −6.13). The slope was also higher in high-luminance trials (slope parameter, γ_*enc*_, Fig. 2*D*, middle; low-luminance = 6.37 [5.21, 7.78] a.u.; high-luminance = 10.32 [8.49, 12.6] a.u., Hedges’ g = −4.51) suggesting a faster rate of sensory encoding in high-luminance trials. Consistent with this, the time to reach peak accuracy was shorter in high-luminance trials (Fig. 2*D*, right; t_peak_: low-luminance = 531 [478, 599] ms; high-luminance = 412 [378, 448] ms, Hedges’ g = 4.86).

The RT measured here represents the duration from the start of the sensory input to the completion of motor response. In order to obtain an estimate of the duration, specifically, of decision-making, we employed the standard DDM approach (Ratcliff, 1978; Ratcliff et al., 2016; Materials and Methods). Briefly, the DDM analyzes the full RT distribution and yields a quantitative estimate of four parameters (Materials and Methods), one of which is t_delay_, a parameter which accounts for the combination of the following: (1) the time taken for the sensory (visual) periphery to transduce and relay information to visual brain areas (i.e., neural response latency); and (2) the time taken for executing the motor response (i.e., motor execution delay). In our tasks, the latter corresponds to the time for the mouse to move its head (and body) to achieve the appropriate nose-touch.

Using this approach, we found that stimulus size as well as luminance had no discernable effect on t_delay_ (two-way ANOVA, size: *p* = 0.308, luminance: *p* = 0.523; interaction: *p* = 0.931; Extended Data Fig. 2-2), and the average value of t_delay_ was 212 ms. Consequently, we estimated the duration of just the sensory encoding stage (temporal integration window) as t_peak_ – t_delay_ = t_peak_ – 212 ms. Across conditions, this took values of 200 ms (412−212 ms; high luminance), 254 ms (466–212 ms; size of 45°), 247 ms (459–212 ms; size of 35°), 281 ms (493–212 ms; size of 25°), and 319 ms (531−212 ms, low luminance).

Thus, conditional accuracy analysis allowed us to quantify the sensory encoding stage in mouse visual perceptual dynamics. We estimated its duration to be brief, varying between 200 and 320 ms across the tested conditions.

Following the completion of sensory encoding, a fully constructed representation of the sensory stimulus is available, as a result of which, additional sampling of the stimulus brings no extra benefits to the performance. Our finding that RTs longer than t_peak_ produce no further increase in conditional accuracy, is consistent with the view (Fig. 2*A*,*C*; see also Extended Data Figs. 2-1, 2-2).

### Stimulus duration and the dynamics of visual decision-making: the memory-dependent stage

The next stage in the time course of perceptual decisions has been identified in human studies as the so-called STM-dependent stage, during which an internal representation of the sensory stimulus is available transiently in memory for guiding behavior (Smith and Ratcliff, 2009). Studies have demonstrated the STM to be labile such that once the stimulus is terminated, sensory information maintained in STM decays and is lost (over seconds; Brown, 1958; Gold et al., 2005; Zhang and Luck, 2009; Barrouillet and Camos, 2012; Ricker et al., 2016).

In our experiments so far, the target stimulus was present on the screen for the full duration of the response window (3 s). Here, to investigate and quantify the STM-dependent stage of mouse perceptual decisions, we performed an experiment in which we shortened the stimulus duration systematically from 3 s to 100 ms. This allowed us to examine the time course of decision behavior following stimulus offset and to examine the shortest stimulus that mice are able to discriminate effectively.

We first examined overall mouse behavioral performance at different stimulus durations. We found that response accuracy was significantly modulated (one-way ANOVA, *p* < 0.001, effect size η^2^ = 0.331; Fig. 3*A*), with accuracy decreasing for shorter stimulus durations (Pearson’s ρ = 0.712, *p* = 0.014). There was also a trend of decreasing median RT for shorter stimulus durations (one-way ANOVA, *p* = 0.056, effect size η^2^ = 0.177; Pearson’s ρ = 0.861, *p* = 0.001; Fig. 3*B*). Additionally, these results revealed, that the shortest stimulus duration needed for mice to be able to discriminate above chance was <100 ms, the smallest duration tested (Fig. 3*A*; see also Extended Data Fig. 3-1).

Next, to examine the decision dynamics following stimulus offset, we aligned trials to stimulus offset, and computed the conditional accuracy. Considering that incomplete sensory encoding may be a confounding factor to the STM decay, we only included those trials on which the stimulus was presented for longer than the duration of the sensory encoding stage, estimated in Figure 2 to be 320 ms.

We observed the classic decay in conditional accuracy with longer RTs (Fig. 3*C*). To quantify the time course of the decay, we fit the conditional accuracy data with a sigmoidal function (Materials and Methods), and estimated three key metrics (Fig. 3*C*; Materials and Methods). The first, peak performance, a_peak_, was 87.3% (median, C.I. = [84.8, 89.9] %), comparable to the asymptotic level of Figure 2, thereby supporting that sensory encoding is, indeed, complete on these trials. The second, the time point at which the conditional accuracy dropped below the peak value, t_decay_, was 469 ms (median, C.I. = [279, 697] ms) after stimulus offset. The third, the first time point at which the discrimination accuracy dropped to a level indistinguishable from the chance, t_chance,_ was 1969 ms (median, C.I. = [1708, 2520] ms) after stimulus offset (Materials and Methods).

Thus, our conditional accuracy analysis allowed us to investigate quantitatively the second, STM-dependent stage in mouse visual perceptual dynamics. We estimated the duration over which above-chance decision accuracy is supported in mice after stimulus offset as ∼1700 ms (i.e., t_chance_ minus the t_delay_).

### The presence of flanker stimulus modulates perceptual dynamics

We next investigated the impact of sensory context on visual decision dynamics. It is well established that the sensory context in which the perceptual target is presented modulates animals’ behavior (Miller, 1991; Meier et al., 2011; Whitney and Levi, 2011). For instance, in the classic flanker task in humans, the co-occurrence of a flanker stimulus with conflicting information can interfere with perceptual performance (Eriksen and Eriksen, 1974; Fan et al., 2002). Recently, similar results were demonstrated in mice using a touchscreen version of the flanker task (You and Mysore, 2020). In this task (Fig. 4*A*), a target grating (always presented at the lower location) was accompanied by a flanker grating at the upper location with either orthogonal orientation (“incongruent” flanker) or same orientation (“congruent” flanker). Compared with the presence of a congruent flanker, the “incongruent” flanker significantly impaired discrimination accuracy (Fig. 4*B*, left; *p* < 0.001, paired-sample *t* test; effect size Hedges’ g = 1.61; re-plotted based on data from You and Mysore, 2020; Materials and Methods). Here, we analyzed that dataset with the conditional accuracy analysis to investigate whether an incongruent flanker affected the sensory encoding stage or the STM-dependent stage of perceptual dynamics.

To investigate the effect of the flanker on perceptual dynamics, we pooled trials from all mice into two groups based on their flanker congruency and sorted the trials based on their RT. Since previous study (You and Mysore, 2020) has demonstrated that the flanker affects performance significantly only when its luminance is higher than (or equal to) that of the target, here we included only high-luminance trials (trials with flanker luminance ≥20.1 cd/m^2^). To examine the sensory encoding stage quantitatively, we followed the approach used in Figure 2 and selected the trials on which mice responded before the stimulus ended (RT < 1000 ms), and aligned them to stimulus onset. Separately, to examine the STM-dependent stage, we followed the approach used in Figure 3 and selected the trials on which responses were made after the stimulus ended, and aligned them to stimulus offset.

The sensory encoding stage was significantly modulated by flanker congruency (Fig. 4*C*,*D*). We found that, the peak conditional accuracy for incongruent trials was significantly lower than that of congruent trials (Fig. 4*D*, left; a_peak_: congruent, median [C.I.] = 87.8 [86.3, 89.6] %, incongruent = 78.5 [76.9, 80.2] %; effect size (congruent-incongruent) Hedges’ g = 11.0), indicating that the presence of a high-luminance incongruent flanker interfered with the sensory encoding of the target stimulus. While the slope parameter for incongruent trials remains comparable to that of the congruent trials (Fig. 4*D*, middle; congruent = 11.9 [9.10, 15.5] a.u., incongruent = 15.3 [11.6, 20.6] a.u.; Hedges’ g = −1.73), the time to reach peak accuracy was, however, shorter for incongruent trials (Fig. 4*D*, right; t_peak_: congruent = 413 [378, 458] ms, incongruent = 374 [340, 410] ms; Hedges’ g = 2.08), consistent with the lower a_peak_ (Fig. 4*D*, left).

The STM-dependent stage also appeared to be modulated by flanker congruency (Fig. 4*E*,*F*). Following stimulus offset, the time at which conditional accuracy dropped to chance was much earlier in incongruent trials than in congruent trials (Fig. 4*F*, right; t_chance_: congruent, median [C.I.] = 2000 [1363, 2000] ms; incongruent = 1145 [816, 1985] ms; Hedges’ g = 2.98). However, this was likely due largely to the lower peak conditional accuracy for incongruent trials (Fig. 4*F*, left; a_peak_: congruent = 84.5 [76.9, 88.6] %; incongruent = 75.9 [70.1, 82.1] %; Hedges’ g = 2.54), as opposed to changes in t_decay_ (Fig. 4*F*, middle; congruent = 299 [197, 1086] ms; incongruent = 343 [126, 802] ms; Hedges’ g = 0.175), or to the rate of decay (slope parameter; data not shown, congruent = −1.82 [−10, −1.0] a.u., incongruent = −4.82 [−10.0, −1.60] a.u.; Hedges’ g = 0.99).

In sum, we found that the presence of an incongruent flanker interferes the sensory encoding stage but not the STM-dependent stage of mouse visual decision dynamics.

### Stimulus onset delay modulates RT distribution but not the CAF

The components of behavioral performance that we have investigated thus far, namely, overall decision accuracy, RT distribution and CAF are related formally in the following way: the overall decision accuracy is the dot product of the CAF and RT distribution.

Our manipulations, thus far, produced changes in the CAF predominantly. Here, we wondered whether task parameters could, instead, alter RT distribution, and possibly do so without affecting the CAF. To test this, we added a delay between trial initiation and target onset (called stimulus onset delay) in the single-stimulus discrimination task. We reasoned that the extent to which mice are unable to adaptively withhold responding could impact the RT distribution.

We found that adding a stimulus onset delay did alter the RT distribution of mice (Fig. 5*A*, upper panel). The median RTs, measured relative to trial initiation, showed an increasing trend with delay (one-way ANOVA, *p* = 0.094; effect size η^2^ = 0.179; Pearson’s correlation = 0.422, *p* = 0.028). This indicated that mice were able to sense the delayed onset of stimulus and thereby withhold their responses. However, mice were unable to withhold responding for the full duration required. By performing a linear regression (Fig. 5*A*, upper panel, dashed line), we found that mice were able to withhold their responses for only 39 ms for every 100 ms of delay. Separately, this increase in RT for longer delays was accompanied by a trend toward lower decision accuracy (one-way ANOVA, *p* = 0.182; effect size η^2^ = 0.132; Pearson’s correlation = −0.358, *p* = 0.067; Fig. 5*A*, lower panel).

By contrast, conditional accuracy analysis revealed no effect of stimulus onset delay either on the sensory encoding stage (Fig. 5*B*,*C*, a_peak_: no-delay, median [C.I.] = 84.2 [82.2, 86.4]%, 200-ms delay = 85.6 [82.9, 89.2]%, effect size (no-delay – 200-ms delay) Hedges’ g = −1.12; slope parameter: no-delay = 7.69 [5.82, 10.7] a.u., 200-ms delay = 6.61 [4.63, 8.74] a.u., Hedges’ g = 0.264; t_peak_: no-delay = 494 [436, 557] ms, 200-ms delay = 552 [476, 680] ms, Hedges’ g = −1.49), or on the STM-dependent stage (Fig. 5*D*,*E*, a_peak_: no-delay, median [C.I.] = 80.5 [75.6, 85.5]%, 200-ms delay = 80.9 [75.6, 84.9]%, Hedges’ g = −0.147; t_decay_: no-delay = 976 [332, 1642] ms, 200-ms delay = 580 [319, 1585] ms, Hedges’ g = 0.877; t_chance_: no-delay = 2214 [1865, 2400] ms, 200-ms delay = 2400 [1935, 2400] ms, Hedges’ g = −1.22).

Taken together, our results from varying the stimulus onset delay show that changes in RT distribution (and overall decision accuracy) are not necessarily accompanied by changes in the CAF. The observed trend of decreased accuracy was accounted for by the fact that with a delay, there were more responses initiated before the sensory encoding was complete, or even before the stimulus was presented (i.e., “impulsive” responses; Fig. 5*B*, histograms). To quantify such impulsivity, we propose an “impulsivity index” (ImpI): ImpI = 1 – average (duration for which mice withhold responses/duration of the delay). Higher positive values of this index indicate greater impulsivity, with ImpI = 1 indicating a complete inability to withhold responding in the face of stimulus delays (maximally impulsive). In the case of our mice, ImpI is ∼0.6 (see also Extended Data Fig. 5-1).

## Discussion

In this study, we quantify two distinct stages in the temporal dynamics of visual perceptual decisions in mice. First, a sensory encoding stage that is subject to the speed-accuracy trade-off, and then, a STM-dependent stage in which decision performance decays once the stimulus disappears. We also demonstrate that the CAF and the RT distribution can be affected independently by experimental manipulations. Whereas stimulus size, luminance, and presence of a foil modulate the CAF with minimal changes to the RT distribution, stimulus onset asynchrony modulates the RT distribution without changes to the CAF. Additionally, our results yield numerical estimates of fundamental psychophysical constants of visual perceptual decision-making in mice. Taken together, this study establishes a quantitative platform for future work dissecting neural circuit underpinnings of the dynamics of visually guided decision-making in mice.

### Estimates of time constants of the dynamics of visual perceptual decision-making in mice

Our results yielded numerical estimates of the duration of sensory encoding (i.e., the window of temporal integration) as 200–320 ms across stimulus size and luminance in mice (Fig. 2). This estimate is similar to that in humans: the internal representation of a visual stimulus is thought to be constructed within the first 200–300 ms of stimulus presentation (Shibuya and Bundesen, 1988; Busey and Loftus, 1994; Vogel et al., 2006; Bays et al., 2011). On the other hand, we also obtained an estimate of the duration of STM as 1700 ms. This constituted the period starting from stimulus offset to the last instant at which responses that are better than chance were initiated (t_chance_ – t_delay_ = ∼1700 ms; Fig. 3*C*). This duration does not necessarily represent just the maintenance of visual stimulus information in STM, it could also represent maintenance of information about the motor response associated with the stimulus (and likely, a combination of the two). Notably, our estimate of the duration of viability of the labile internal representation in mice falls in the same range as has been reported from human studies (Sperling, 1960; Posner and Keele, 1967; Phillips and Baddeley, 1971).

We have interpreted the decay in performance following stimulus offset as being because of loss of information in STM. A potential confounding factor to this interpretation is differences in the internal state of the animal, in selective attention, or more generally, task engagement. It is possible, for instance, that all the trials with longer RTs represent those in which mice did not pay attention to the stimulus (or more generally, were disengaged from the task), thereby being associated with lower accuracy. We believe this unlikely because attention/engagement was not varied systematically, here (unlike in the flanker task; Fig. 4). Even if loss of attention or engagement were a factor, any improvements in conditional accuracy because of increased attentiveness or engagement would only lengthen STM. From this perspective, our estimate of 1700 ms serves as a lower bound for the duration of STM.

This estimate of 1700 ms also represents a lower bound for working memory (WM). Whereas STM refers to the retention of information even when it is not accessible from the environment, WM refers additionally to the ability to manipulate this information and protect it from interference (Cowan, 2008; Postle and Pasternak, 2009). WM can be lengthened with training. For instance, in tasks that require animals hold information over an enforced delay period before responding, it has been reported that mice can learn to perform well with delay periods up to 5 s (Liu et al., 2014). Here, by allowing the natural evolution of the dynamics of decision-making to occur without an imposed delay period, we were able to estimate the “intrinsic” (lower bound for the) duration of STM.

### Estimates of the operating range of stimulus features for visual perceptual decision-making in mice

This study also yielded estimates for the range of values of various stimulus features within which mice are able to discriminate the visual target. The smallest stimulus size and lowest luminance (tested) at which mice were able to discriminate orientation above chance were 25° and 2.00 cd/m^2^, with mice performing at >80% accuracy for most luminance values at that smallest size. The shortest stimulus that mice are able to discriminate above chance was ≤100 ms (Fig. 3*A*). Further, based on the x-intercept of the CAF in sensory encoding stage (median [C.I.] = 236 [215, 253] ms, pooling all trials of various sizes and luminance from Fig. 2), we were able to refine this estimate to be ≤53 ms (conservatively, after subtracting t_delay_ = ∼200 ms). This is consistent to the estimation (40–80 ms) from a previous study based on visual cortical activity (Resulaj et al., 2018). In a subgroup of animals (*n* = 3), we tested whether mice are able to discriminate orientation of the target stimulus (25°, 0.1 cpd, 16.2 cd/m^2^) when it was 50 ms long. Two out of the three mice showed a response accuracy higher than chance (accuracy = 57.9%, 210 correct out of 363 trials, *p* = 0.002, binomial test; and 55.6%, 143/257, *p* = 0.040, respectively), consistent with this refined estimate. These findings that mice are able to discriminate visual stimuli in demanding sensory contexts suggest that the visual perceptual abilities of mice may be underrated.

The best discrimination performance reported in mice (accuracies >90%) have typically been obtained using large, often full-field, grating stimuli (Andermann et al., 2010; Long et al., 2015). In our single target discrimination task, the best performance ranged lower, between 75% and 90% (Fig. 1*C*), consistent with our use of “small” stimuli (relative to those typically used in mouse vision studies; Prusky et al., 2000; Prusky and Douglas, 2004; Wong and Brown, 2006; Busse et al., 2011; Long et al., 2015). Indeed, in our pilot study, the performance plateaued at ∼93% for a stimulus size ≥45° (Extended Data Fig. 1-1*C*,*D*). These results suggest that full-field stimuli may be effectively replaced by 45° stimuli to achieve best performance levels.

The best discrimination performance exhibited a dip at the highest luminance (Fig. 1*C*). This is potentially well accounted for by signal saturation: because the visual system adapts to the relevant range of stimulus luminance for best encoding (Ohzawa et al., 1982), the interleaved presentation of stimuli with different luminance can render the maximum-luminance stimulus unfavorable because of signal saturation (Long et al., 2015). Consistent with this idea, when the maximum-luminance stimulus (25°, 0.1 cpd, 130 cd/m^2^) was presented *alone* in block design (Extended Data Fig. 1-1*C*, the black box at the left most, group median [C.I.] = 85.7 [77.6, 92.1] %), response accuracy was nominally higher than when it was presented interleaved with stimuli of varying luminance (Fig. 1*C*, the red box at the right most, group median [C.I.] = 79.7 [61.9, 91.9] %). These results indicate that a good upper bound for stimulus luminance in mouse experiments may be ∼34 cd/m^2^.

### Stimulus and task parameters modulate perceptual performance through a variety of mechanisms

Increase in stimulus size and luminance both improved the overall discrimination performance of mice (Fig. 1). However, analysis of conditional accuracy revealed that whereas increasing each increased the peak conditional accuracy (a_peak_), only increasing the stimulus luminance increased the slope of the CAF and resulted in a shorter t_peak_ (Fig. 2). We propose that these differences in the CAF reflect differential mechanisms at one or more levels of the underlying sensory processing. Specifically, in our experiments, varying stimulus luminance (through varying the intensity of just the bright phase of the grating) also varied stimulus contrast (relative to the dark background). On the other hand, increasing stimulus size increased the total luminance without affecting contrast. Consequently, differential activation of lateral inhibitory mechanisms for spatial contrast may account for the observed differences in CAFs. Separately, whereas increasing stimulus size and luminance both increase the total number of photons impinging on the retina, increasing stimulus size would activate a broader spatial distribution of photoreceptors (at a fixed signal-to-noise ratio), while increasing stimulus luminance would cause a largely fixed group of photoreceptors to receive a higher density of photons (and higher signal-to-noise ratio). Consequently, differential mechanisms of sensory integration (of the two) may also account for the observed differences in CAFs.

Separately, manipulating attention (by presenting a flanker) and manipulating the stimulus onset asynchrony both caused a reduction in response accuracy (Figs. 4*B*, 5*A*, lower panel). However, again, the analysis of conditional accuracy suggests that the mechanisms underlying the two are different: the capture of attention by the flanker interferes with the target’s sensory encoding, whereas adding a prestimulus onset delay results in change of the RT distribution without affecting the CAF.

Taken together, our results demonstrate that although manipulating stimulus parameters or experimental conditions may induce seemingly similar changes in perceptual performance (overall accuracy), their underlying mechanisms could be different. The conditional accuracy analysis serves as an informative tool to explore these mechanisms and to understand the dynamics of perceptual decision-making.

### Qualitative differences between stimulus features as well as between task-difficulties

Across the various tasks and stimulus conditions that we studied here in mice, the sensory encoding stage ended rapidly, around 300 ms. However, in a recent study in which rats discriminated the direction of motion of a patch of random dots, the sensory encoding stage continued through at least 1.5 s (the longest RT bin reported; Shevinsky and Reinagel, 2019). We propose that this difference in the duration of sensory encoding may be because of fundamentally different nature of stimulus features used in these two studies. Consistent with this proposal, a study on human visual psychophysics (Burr and Santoro, 2001) has reported a temporal integration window of 200–300 ms when stimulus contrast of a patch of random dots was varied (similar to our results in mice), but a substantially longer integration window of 3 s when their motion coherence levels were varied (similar to the above-mentioned results in rats).

Separately, our results also highlight that “task difficulty” may be altered in qualitatively different ways, producing distinct outcomes on behavior. In the literature, task difficulty is often increased by making target stimuli more ambiguous or by introducing distracters (which we did also). Such manipulations often cause subjects (animals) to respond slower, allowing them time to gather more information to produce better performance (which we found, as well). However, when we shortened stimulus duration, which can plausibly be considered to also increase task difficulty, we found the opposite result, mice responded faster as the target stimulus became shorter (Fig. 3*B*). This potentially counterintuitive effect (faster RTs for a “more difficult” task) is explained well by the conditional accuracy analysis (Fig. 3*C*). Whereas shortening the stimulus duration makes the task more difficult, responding more slowly to shorter stimuli does not grant a perceptual benefit to the animals: once the stimulus has disappeared, withholding responses for longer would only increase the risk of losing information owing to memory decay. In other words, short stimuli impose a “time pressure” on animals to make decisions quickly. Thus, task difficulty may be altered in qualitatively different ways, with distinct behavioral effects.

### Optimal sensory sampling during visual perceptual decision-making in mice

An intriguing observation in our study is that across tasks, the peak of RT distribution (the RT bin with the largest number of trials) seemed to always occur around t_peak_ (Figs. 2*A*,*C*, 4*C*). Since the RT distribution can vary independently of the CAF (as demonstrated in Fig. 5), there is no a priori reason that the peak of RT distribution and the t_peak_ must change together. We propose that responding with RTs close to t_peak_ may be an optimal behavioral strategy for the mice. As indicated by the CAF, mouse response accuracy increased as RT increased until it reached a plateau at t_peak_. Responding earlier than t_peak,_ therefore, would sacrifice accuracy, while responding later than t_peak_ would needlessly delay response (reducing the reward rate). Consequently, responding with the peak of RT distribution being equal to t_peak_ would be optimal. Testing this optimality hypothesis would require future experiments to manipulate the temporal integration window (t_peak_) substantially (much more than the 40- to 120 ms change we find in Figs. 2*B*,*D*, 4*D*), for instance, by manipulating stimulus coherence (Burr and Santoro, 2001) or the volatility of environment (Piet et al., 2018), and to ask whether this is accompanied by a commensurate shift in peak RT.

## Synthesis

Reviewing Editor: Nicholas J. Priebe, University of Texas at Austin Decisions are customarily a result of the Reviewing Editor and the peer reviewers coming together and discussing their recommendations until a consensus is reached. When revisions are invited, a fact-based synthesis statement explaining their decision and outlining what is needed to prepare a revision will be listed below. The following reviewer(s) agreed to reveal their identity: Andrea Benucci.

Synthesis

Two reviewers evaluated the manuscript “Dynamics of visual perceptual decision-making in freely behaving mice” and both recognized that this work could provide an important foundation for applying psychophysical techniques to freely moving mice. There was consensus, both in the reviews and in the consultations that followed, that while the manuscript in its present form is a good start, there are a number of issues. Reviewers raised significant concerns related to the form of the visual stimulus employed, the statistical analyses performed to quantify performance, the pooling of data across animals, and the motivation for the use of the drift diffusion model. The manuscript could be streamlined by integrating or removing the supplementary figures into the main document. The reviewers provide extensive comments which you should find useful in revising your manuscript, if you decide to resubmit it to eNeuro or to another journal.

----------

REVIEWER 1

ADVANCES IN THE FIELD

I have to read the reply to my comments to judge with confidence whether or not this study provides advances in the field.

COMMENTS

The authors of the manuscript “Dynamics of visual perceptual decision-making in freely moving mice” examined the behavior of mice trained in visual discrimination tasks while varying several stimulus features such as stimulus size, contrast, duration, onset delay, and presence of a foil (flanker or distractor). Using a conditional accuracy function (CAF, correct rate conditional to reaction times, RT), they assert that two stages for the decision making could be identified, similar to what found in human studies: a sensory encoding stage with a typical speed-accuracy tradeoff, and a short-term memory (STM) stage with a typical decay after stimulus offset.

As remarked by the authors, I agree this work presents an interesting set of observations about “fundamental psychophysical constants of visual perceptual decision-making” and it provides a quantitative platform for future work on the underlying neural circuits. It is also interesting to compare behavioral responses to different task within the same experimental setup and stimulus modality.

However, I have a few questions and observations on the Methodology and on the Results that I believe should be addressed.

1) A main methodological concern is about the stimulus contrast: as stated at Ln 32, the Michaelson contrast was computed by varying the luminance of the bright phase of the grating, while keeping the “black phase” at the same dark luminance of the background. This is unconventional: luminance and contrast are typically varied independently by modulating both the dark and bright phases of the grating around a set mean luminance. By not doing so, all observations about changing the stimulus “contrast” are here conflated with changes in stimulus luminance. This creates a significant interpretability problem for almost all results presented. For instance, Fig. 1C,D show a clear dip at high “contrasts", discussed briefly (too briefly imo) at Ln 681 in terms of contrast adaptation and signal saturation. It may be that very bright stimuli are somewhat “aversive” to mice, thus setting animals in a different behavioral state, for example. All results based on “contrast” suffer from this conflation.

2) Related: is the effect shown in Figure 2E, where higher contrast (and luminance) reduces RTs (because the “rate of sensory encoding was faster for high contrast stimuli", Ln371) the same phenomenon seen in Fig. 1E, but linked to changes in stimulus size? Both manipulations increase the overall luminance (see comment above). It is also unclear why median RTs do not depend on contrast in 1E, but t_peak values do in Fig. 2E. If the “rate of sensory encoding was faster for high contrast stimuli", why this is not the case in Fig. 1E? Similarly, median RTs seem to be affected by stimulus size in Fig. 1E, but t_50 and t_peak are not in Fig. 2C; why so? Granted that median RTs and t_peak are different quantifiers, it is however the case that the “RT distribution was centered around t_peak (Fig. 2ABD, 4C)", Ln 693. Apologies if I missed a fundamental methodological distinction.

3) Another methodological concern is about the calculation of the conditional accuracy curves. As stated at Ln 205, trials from all mice were pulled together as if they were from one single mouse. However, if, for example, each mouse had a different conditional accuracy function, (e.g., an inverted U-shape curve), with variable “widths” along the RT axis, then pooling these curves together could produce a seemingly plateauing curve with a dipping trend at large RTs, as observed in Fig. 2A. To address this, the authors could show CAF curves also for individual mice.

4) Related, the authors discuss that “across tasks, mice responded with nearly constant RT” (Ln 687), referring to Figures that do not show individual animal behaviors and associated variability. Apologies if I misunderstood this part.

5) CAF curves are computed by fitting an asymptotic function to binned data points. However, the authors do not compare fitting parameters across conditions, but rather define new quantities on the fitted curves (a_peak, t_50, t_peak): why so? As the authors mention at Ln 220, in any case their measures depend on the fitting parameters.

6) It would also be interesting to see how CAF fits depend on the binning size. For instance, in Fig. 2A it seems that only 3 or 4 data points constrain the fit (those on the rising slope), therefore changing the binning could significantly affect t_50 and t_peak values. This seems particularly striking in Fig. 4C: there, the data points show no difference till about 400 ms, but the fits start diverging at and before 400 ms. This leads to reported significant changes in panel D that do not seem to be supported by the data, where “a_peak” and “t_peak” (guessed from the data points) seem very much the same across congruency conditions.

7) The authors also use a drift-diffusion model. Why so, and what results depend on this modeling effort (besides Ln 320) is unclear. The behavioral paradigm used here does not seem to require any accumulation of evidence. Even at 20% “contrast", stimuli are extremely salient. Furthermore, when using DDM to explain the lack of RTs contrast modulation (but with a dependence on stimulus size) the authors point to a “complementarity” between starting point and drift rate. This observation seems to be statistically supported only for the largest stimulus size. If I am not misreading the stats, then I would suggest downplaying this part of the results. In my opinion, the overall constancy of RT rather indicates that a DDM is not a good modeling framework for these tasks.

8) I failed to fully grasp the motivations for the memory-dependent stage. At Ln 419 this analysis is introduced as to “test for the existence of the second ‘decaying’ stage"; how could this stage not exist? Its absence would imply an organism that cannot forget, even long after a stimulus has been removed. I would suggest rephrasing the motivations, unless I largely misunderstood this part.

9) In Figure 3D, histogram count, it seems indeed that only in a reduced number of trials, animals responded after stimulus offset. Isn’t it conceivable that, rather than an STM stage, these trials reflect a different ‘internal state’ (e.g., of attention, arousal) of the animal? Furthermore, one would guess that when considering trials with durations longer than 332 ms and responses both before and after stimulus offset, the resulting CAF curves should have an inverted U-shape. If so, it would be interesting to show this ‘summed’ behavior.

10) Still concerning the STM stage, I failed to understand the effect shown in Fig. 4E. From panel C we learn that when the animal makes a choice while the stimulus is present, the presence of a ’distractor’ lowers %correct. This should be true (possibly even more so) when the choice is made after the offset of the stimulus. The rate of decay may be unaffected by the flanker, but the vertical separation between the red and blue curves should.

11) For the analyses in Figure 4 the authors collapsed across flanker contrasts. Is it the case that the few effects reaching significance are associated only to high contrast/luminance conditions?

12) Figure 5, I appreciated the effort to separate the components of the “dot-product” that results in the overall decision accuracy. However, in consideration of the “ascending sequence” approach (Ln 135) and 0.6 impulsivity, I am wondering if the only message here is that animals get frustrated for the longer waiting times, which accordingly does not impact sensory perception nor STM components but produces a trend for lower %correct. If so, then this part could be significantly shortened. However, I found it interesting that mice withheld responses for about 40 ms for every 100 ms of delay (Ln 574); is this related to the other observation that the shortest stimulus duration which produced better than chance accuracy was also 40 ms? Some sort of minimal window for the integration of sensory information that determines a temporal “discretization” in the state-action plan?

13) Finally, I have a comment about a discussion point, Ln 686, regarding the optimality argument. This point is potentially interesting, but could the authors try to back it up with some quantifications? For instance, could they simulate alternative RT distributions (of somewhat meaningful shape) against which to test their optimality hypothesis? Otherwise, the trivial interpretation here might be that RT bins with most trials have also many correct trials-because animals have good performance.

14) Minor points:

a. Ln 249, permutation tests, have the authors used balanced datasets across conditions?

b. In several parts across the manuscript the references to supplementary information change format.

c. In a few parts it is unclear how the statistical tests have been used; e.g., Ln 443 I was expecting a regressive test (Spearman?) for the trend “of decreased RT as the stimulus duration decreased” instead the test used was a 1-way anova.

d. It was not always easy to understand what was kept constant and what varied across experiments, with this information spread around main text, captions, and methods. I would suggest finding a way to graphically include this information in figure panels.

e. It would help the comparison across tasks if CAF panels had consistent x- and y-ranges.

f. For Discussion, it would be interesting to read about agreement (or not) between this work and another very relevant study (not cited) by Shevinsky and Reinagel (Frontiers in Neurosci. 2019).

----------

REVIEWER 2

ADVANCES IN THE FIELD

The current study applies a set of robust psychophysical approaches to assess the temporal constraints of sensory integration in the mouse. These will be important baseline data for the investigation of the underlying circuits.

COMMENTS

The mouse visual system has become a premiere model for understanding the circuits supporting sensory integration and decision-making. Thus, quantitative assessments of the different time constants of integration will be important for interpretation of studies probing these circuits. The current study applies a set of robust psychophysical approaches to fill this gap and define key quantitative constraints on durations of the minimally and maximally useful stimulus, sensory integration, and short-term memory. Overall, this is a strong study that will be of interest to the mouse vision community. However, I do have a number of suggestions that will increase the clarity of the manuscript.

1. The choice to use signal detection theory to calculate d’ and c with a two-alternative forced choice task is an unusual one given that in these tasks percent correct is directly related to sensitivity (there is no need to calculate d’) and they are by definition

"criterion-free”. This is clear from the strange approach that the authors used to define FA rate (as the miss rate for one of the two orientations). Thus, it seems like the measure of c reflects a choice bias rather than a criterion? I think it would be more transparent to simply present this as percent correct and choice bias rather than imposing a signal detection framework on these data.

2. It is surprising that the drift-diffusion model finds that increases in contrast change the starting point in addition to the drift-rate. My understanding is that an imbalance in starting point reflects a choice bias- however, the mouse should be unaware of the upcoming stimulus contrast (and the authors showed that there is minimal effect of contrast on choice bias). More clarification of this result is needed. Also, given that the conclusions of the drift-diffusion model (such as the t-delay) are central to many of the arguments, it would make sense to incorporate these data into the main figures.

3. Size, contrast and flankers each decreased percent correct, but had different effects on the duration of sensory encoding. Decreasing size had no effect, decreasing contrast increased the time to peak (without an effect on the time to 50%), and incongruent distractors increased the time to 50% and decreased the time to peak. More discussion is needed to understand the interpretation of these disparate results of difficulty on decision time.

4. The authors find a positive relationship between stimulus duration and reaction time (Figure 3C); this is surprising given that shorter durations should be more difficult and could benefit from some discussion.

5. Why is there no difference in peak % correct during the shortest RTs on congruent/incongruent trials (Figure 4E)? Shouldn’t these be offset from each other?

6. The authors provide a reasonable qualification of their short term memory result in the discussion- that it might reflect more global, “attentive” state on these long RT trials (where the sensory information was never encoded) than the loss of the encoded information. However, I’m not sure that their reference to the flanker experiment rules out the possibility of this interference, due to the multiple potential meanings/interpretations of “attention”. Namely, the flanker likely affects task difficulty or a form of selective attention, whereas the concern about the long RT trials would reflect drifts in engagement or arousal that are not probed by the flanker task.

7. The authors are missing some key studies of temporal integration in the literature: Histed and Maunsell, 2014 and Resulaj et all., 2018.

8. The title on line 473 should be “Task-irrelevant foil”.

9. On line 661, the authors say the mice were able to discriminate durations as short as 40 ms. However, the shortest stimuli tested were 100 ms. I think the 40 ms duration is one calculated from the t-delay.
